# Normatively irrelevant disgust interferes with decision under uncertainty: Insights from the Iowa gambling task

**DOI:** 10.1371/journal.pone.0306689

**Published:** 2024-08-01

**Authors:** Giulia Priolo, Marco D’Alessandro, Andrea Bizzego, Laura Franchin, Nicolao Bonini

**Affiliations:** 1 Department of Psychology and Behavioural Sciences, Aarhus University, Aarhus C, Denmark; 2 Department of Psychology and Cognitive Science, University of Trento, Rovereto, Italy; 3 Neuraptic AI, C., Madrid, Spain; 4 Department of Economics and Management, University of Trento, Trento, Italy; University of North Texas Health Science Center, UNITED STATES OF AMERICA

## Abstract

This study investigates whether a not informative, irrelevant emotional reaction of disgust interferes with decision-making under uncertainty. We manipulate the Iowa Gambling Task (IGT) by associating a disgust-eliciting image with selections from Disadvantageous/Bad decks (Congruent condition) or Advantageous/Good decks (Incongruent condition). A Control condition without manipulations is also included. Results indicate an increased probability of selecting from a Good deck as the task unfolds in all conditions. However, this effect is modulated by the experimental manipulation. Specifically, we detect a detrimental effect (i.e., a significant decrease in the intercept) of the disgust-eliciting image in Incongruent condition (vs. Control), but this effect is limited to the early stages of the task (i.e., first twenty trials). No differences in performance trends are detected between Congruent and Control conditions. Anticipatory Skin Conductance Response, heart rate, and pupil dilation are also assessed as indexes of anticipatory autonomic activation following the Somatic Marker Hypothesis, but no effects are shown for the first two indexes in any of the conditions. Only a decreasing trend is detected for pupil dilation as the task unfolds in Control and Incongruent conditions. Results are discussed in line with the “risk as feelings” framework, the Somatic Marker Hypothesis, and IGT literature.

## Introduction

The neo-classical conception of the human being as a rational decision-maker has been challenged by the rising evidence on the role of affective and emotional reactions in the decision process stemming from psychology and neuroscience. Theories such as the “Dual process theory”[1–6], “risk as feeling” [7], and the Somatic Marker Hypothesis (SMH) [8–10], suggest that mental images of stimuli and events are marked in people’s mind with positive or negative affective reactions that occur automatically and before overt reasoning on declarative knowledge and can be experienced mostly unconsciously as feeling states. These affective reactions are then used as informative elements that guide the decision process through an implicit strategy which leads people to approach something they like and avoid something they dislike (cfr.“Affect heuristic” [7,11,12]).

Moreover, according to the SMH, the generation of these emotional tags (“*somatic markers*”) might be associated with autonomic neural activation and can be detected as anticipatory physiological arousal (e.g., by measuring anticipatory skin conductance response, aSCR). For example, Bechara and colleagues [8,13,14] found that patients with damage to the ventromedial prefrontal cortex, a brain region involved in the decision process, could not act advantageously in the Iowa Gambling Task (IGT) [8,15] nor showed any kind of anticipatory physiological response.

The IGT is a widely used behavioral task that mimics real-life decision-making in conditions of uncertainty. The goal of the IGT is to maximize an initial endowment of play money (2000 $) by picking cards from four decks. Each deck is associated with a fixed gain and unpredictable losses: two decks (i.e., Bad decks, Deck A & B) yield a high gain (100 $) and high losses, thus choosing from those decks is riskier and disadvantageous in the long run; the other two decks (i.e., Good decks, Deck C & D) instead yield a smaller gain (50 $) but also smaller losses, thus choosing from those decks is safer and advantageous in the long run. Better IGT performances are usually associated with more frequent selections from the Good decks. The IGT simulates an uncertain situation since participants are informed about the goal of the game, but they do not know the number of selections they will have to make (which is 100) nor the internal win/loss schedule of the decks. In Bechara and colleagues’ studies [8,13,14], non-impaired control subjects were found to perform more efficiently (i.e., increase selections from the Good decks) than injured ones as the task unfolded, and to generate aSCR while pondering to choose from the Bad decks. It seems that the anticipatory affective reaction—associated with the risk contingency of the Bad decks and experienced as a somatic state, helped participants to avoid those decks and thus pursue a safer performance (e.g., by alerting them of the potential threat, even before they consciously understood the riskier nature of the decks [9,16,17]). Similar results have been detected also in other non-clinical samples, although it is important to note that different, yet effective, strategies were also adopted by healthy populations. A review from Steingroever et al. [18] for instance, shows consistent across and within-group variability in IGT performance in non-clinical populations and suggests that some healthy participants’ performance might rather focus on minimizing the frequency of losses (i.e., choosing more from Decks B and D, which entail higher but less frequent losses), than maximizing long-term outcomes (i.e., choosing more from the Good decks) as originally assumed by Bechara et al. (a phenomenon also known as Prominent Deck B, [15,19–21]).

Overall, relying on one’s feelings can often be an effective strategy to navigate complex and uncertain situations, although in some circumstances it can be disruptive. This proved to be especially true when our feelings are deliberately manipulated [7,9]. For instance, Priolo and colleagues [22] associated the hearing of an unpleasant sound with selections from the IGT’s decks. This manipulation was expected to make participants experience a normatively irrelevant (i.e., not informative and not useful to the overall task goal), negative affective reaction towards Bad or Good decks that should have interfered with their performance. Results showed a detrimental effect of the manipulation (i.e., a systematic reduction in selection from the Good decks) when the unpleasant sound was associated with the Good decks (i.e., Incongruent condition) compared to a condition with no manipulation (Control). No effect of the manipulation was instead found when it was associated with the Bad decks (i.e., Congruent condition) since no significant decrease in selections from these decks was observed compared to the Control condition. Nevertheless, in Priolo et al. [22] the unpleasant-sound experimental manipulation generated only a generic, not emotional, affective reaction of unpleasantness. It is possible that more intense, discrete emotional responses (e.g., fear, disgust, anger), might be more effective in influencing people’s behaviors (i.e., lowering selections from the manipulated decks) in both conditions, thus eliminating the asymmetric effect found in Priolo et al. [22] study. A study by Pittig et al. [23] indeed, showed an effect of a fear-inducing manipulation when it was associated with both Good and Bad decks. In this study, the authors associated spider images with the decks, creating two experimental conditions similar to the ones used in Priolo et al. [22]. Results showed that participants with spider phobia avoided selections from the manipulated decks in both “Non-conflict” (i.e., Congruent) and “Conflict” (i.e., Incongruent) conditions leading them to safer performances in the first case and to riskier ones in the latter. The detrimental effect of the fear-inducing manipulation in the Incongruent condition was also confirmed by studies using pictures of angry faces with clinical and sub-clinical socially anxious participants [23,24]. A corresponding Congruent condition was not included in those studies. However, in Pittig et al. [23] studies, the fear-inducing manipulation, although normatively irrelevant as the one used in Priolo et al. [22] was meaningful to the participants since it was related to their clinical and sub-clinical condition. Overall, these results are in line with previous studies highlighting carry-over effects of incidental emotions, usually experimentally induced via mood-induction procedures (e.g., video clips) on people’s unrelated judgments and decisional processes under risk and uncertainty [25–30]. However, results were found to be moderately heterogeneous and mostly related to studies assessing fear, anger, sadness/unhappiness, or broad valence [27]. Thus, the necessity to further investigate the effect of other specific emotions (e.g., disgust) has been recently raised [27].

In the present study, we thus further investigated the role of a normatively irrelevant, negative emotional reaction of disgust in shaping risk-taking behaviors in a manipulated version of the IGT. Specifically, we designed our disgust-eliciting manipulation (i.e., the presentation of a highly disgusting image) to elicit an intense, negative emotional reaction as in Pittig et al. [23] while being normatively irrelevant and unrelated to any clinical meaning or association to danger as in Priolo et al. [22].

Disgust-eliciting manipulations were found effective in shaping several decision-making processes in previous studies, e.g. increasing acceptance rates in the Ultimatum Game [31], eliminating the endowment effect [32] and the status quo bias [33], and decreasing consumers’ product evaluation [34]. The influence of disgust on IGT performance has been assessed in previous studies, but only in terms of incidental disgust induction before task administration. For instance, Heilman et al. [35] exposed participants to a disgust-inducing video clip before the IGT and showed that participants who adopted a reappraisal self-regulation strategy tended to be more risk-seeking compared to participants who did not regulate their emotions. On the contrary, Bollon & Bagneux [36] found that participants exposed to a video clip to induce a certainty-associated emotion of disgust performed more advantageously compared to participants who were instead exposed to a video inducing an uncertainty-associated emotion (e.g., fear or sadness, see also [37,38]). To the best of our knowledge, no prior study associated a disgust manipulation directly to card selection as in our design.

The role of disgust in shaping people’s risky choices is also congruent with new perspectives on risk-taking that integrate psychology and evolutionary biology, and describe the emotional reaction of disgust as a mechanism for calibrating decisions under risk [39]. According to this view, disgust has been shaped by evolution to play a pathogen-avoidance function which involves a tradeoff between a potential cost (e.g., being exposed to a contaminated substance) and a potential gain (e.g., feeding or mating). In these terms, disgust propensity can be seen as a risk-averse strategy, and associations between higher disgust propensity and lower risk-taking behavior have been found [39], although using self-reported measures. Thus, investigating the role of disgust on real risk-taking behaviors such as in the one simulated in the IGT, would constitute a relevant improvement in helping to shed light on this under-investigated topic.

In the present study, we thus wanted to investigate whether a normatively irrelevant emotional reaction of disgust driven by a disgust-eliciting image, might have a detrimental effect (i.e., lowering selections from the Good decks) when it is associated with Good decks (Incongruent condition), while having instead a beneficial effect (i.e., increasing selections from the Good decks) when it is associated with Bad decks (Congruent condition) compared to a Control condition with no manipulation. In line with the studies and theoretical frameworks discussed above [22–24,40], we expected that, in the *Incongruent* condition, the emotional reaction of disgust associated with the Good decks through the manipulation would detriment participants performance (i.e., participants would avoid more the advantageous Good decks) compared to the *Control* condition, especially in the initial stages of the task, when the risk contingency of the decks is still highly unclear (H1). Vice-versa, in the *Congruent* condition, we expected that the emotional reaction of disgust associated with the Bad decks would improve participants’ performance (i.e., participants would avoid more the disadvantageous Bad decks) compared to the *Control* condition, again in the initial stages of the task (H2). This last expected finding would be in line with Pittig et al. [23] but in contrast with Priolo et al. [22] in which an affective, less intense unpleasant reaction was induced.

Nevertheless, we expected to find a learning effect across conditions (H3). Classical findings in IGT studies [8,13–15] indeed, showed that non-impaired participants gradually learned that choosing from the Good decks is the safest strategy to reach an overall better performance and thus increase their rate of selections from those decks as the task unfolds. This classical learning trend was also confirmed in all conditions in Priolo et al. [22] and Pittig et al. [23].

In line with the SMH aSCR, heart rate (HR), and pupil dilation were also assessed in the present study as indexes of anticipatory autonomic activation. Original studies from Bechara and colleagues [13,41,42] found an association between better performances in the IGT and the generation of aSCR preceding selections from the Bad decks in non-clinical participants. Significant associations and differences before selections from the Bad and the Good decks were also found in recent metanalyses [43,44], although the effect was small to medium. A study from Crone et al. [45] furthermore, reported an association between HR deceleration before selections from the Bad decks and better performance on the IGT, but results were not supported by Hayes & Wedell [46], Miu et al. [47] and Priolo et al. [22]. In the latter study, for instance, deceleration in HR was found before selections from the Good decks potentially indicating the activation of a *positive* somatic marker that motivated participants to pursue the advantageous strategy of picking more from those decks. Besides, Simonovic et al. [48] reported an association between poorer overall performance and increased pupil dilation on the last pick from a Bad deck, as well as between better overall performances and increased pupil dilation for Good decks’ last selection, thus suggesting the generation of different somatic markers for Good and Bad decks. Nevertheless, studies investigating the role of HR and pupil dilation are still scarce and results are mixed. To the best of our knowledge, no previous study integrated both of those measurements with aSCR in an IGT study.

In line with the SMH, we expected that our manipulation would impact participants’ somatic markers development. Specifically, we hypothesized that in the *Incongruent* condition, the mismatch between the riskier contingency of the Bad decks and the disgust-eliciting image associated with the Good ones, would mislead participants’ somatic marker system, preventing them from developing a clear somatic-alert-sign towards Bad decks. Thus, we expected to find an interaction between the condition and deck choice where in the *Congruent* condition the autonomic activation prior to selections from Bad decks would be higher compared to Good ones, whereas in the *Incongruent* condition the autonomic activation would be lower, or the same, for selections from Bad decks compared to Good ones (H4). In particular, the former finding would be due to the joint effect of the relevant negative affective reaction driven by the Bad decks’ risk contingency together with the non-relevant disgust-eliciting image associated with the same decks.

## Methods

### Participants

140 adults participated in the study and were reimbursed up to 10€. Six participants were excluded due to technical issues (*n* = 1) or already knowing the task (*n* = 5). Thus, the final sample included 134 participants (54.5% female, *M*age = 24.06 years, *SD* = 4.82), randomly assigned to one of three conditions.

### Design and procedure

Data collection took place from 25 January 2021 to 26 May 2021 at the Department of Psychology and Cognitive Sciences, University of Trento (Italy). The study design and procedure were adapted from Priolo et al. [22]. Three between-subjects conditions were created: 1) a *Control* condition with no manipulation (i.e., standard IGT, *n* = 46); 2) a *Congruent* condition (*n* = 46) in which the Bad decks were associated with a disgust-eliciting image, while Good decks were not manipulated; 3) an *Incongruent* condition (*n* = 42) in which the Good decks were associated with a disgust-eliciting image, while Bad decks were not manipulated.

The experiment was conducted in individual sessions of 90 minutes and took place in a mild, constantly illuminated room. Stimuli were presented on a full HD monitor screen (23 inches) placed about 60 cm away from the participant.

First, written informed consent to partake in the study was obtained from all participants. Next, sensors for the acquisition of electrodermal activity (EDA) and photoplethysmograph (PPG) were placed, and an eye-tracker five-point calibration was performed. Then, physiological baseline activity was recorded for five minutes while participants were asked to relax. Subsequently, classical IGT instructions [8] (i.e., including a hint about the presence of advantageous decks) were given, and participants performed the task according to their condition. To maintain participants’ engagement during the task, they were told that their final reimbursement would have been calculated based on their performance up to a maximum of 10€. Finally, they were debriefed and reimbursed. Participants’ impulsivity traits (using the BIS/BAS scale [49]), negative state mood (pre and post-test, using the PANAS scale [50], and demographics were also assessed (see Supporting Information for details).

The study received approval from the Research Ethics Committee of the University of Trento, (protocol number: prot_2019_004), and the Declaration of Helsinki’s principles were followed.

### Materials

A computerized version of the IGT was administered via an OpenSesame 3.2.8 program [51]. The original gain and losses schedule [8] was used and the game was automatically stopped after 100 trials. The four decks were presented on screen in grayscale to maintain constant luminance and labeled 1, 2 (Bad decks), 3 and 4 (Good decks). Decks’ position was held constant throughout the game. The virtual endowment amount (2000€) updated after each trial was shown above the decks.

Each trial consisted of 1) “Relax section” (5 sec), the word “Wait” appeared under the decks. Participants were instructed to relax and not to think about anything. This section served as an inter-trial interval (ITI) to allow physiological indexes to return to baseline. No actions were allowed at this time; 2) “Think section” (5 sec), the word “Think” appeared under the decks. Participants were instructed to think from which deck they would have picked next. No actions were allowed during this time; 3) “Selection section”, the word “Choose” appeared under the decks. Participants were instructed to pick a card by pressing the corresponding number on the keyboard. No time constraints were given to make the choice; 4) “Response section” (3 sec), the chosen deck was highlighted in white scale while the others remained in the background in grayscale. In *Congruent* and *Incongruent* conditions, the disgust-eliciting image was shown on the deck if respectively a Bad or a Good deck was chosen. The image (Number: 1138; Category: body product; Disgust: 6.92; Valence: 2.07; Arousal: 4.3; Luminance-matched) was selected from the DIsgust-RelaTed-Images [52] database (available at: http://dx.doi.org/10.5281/zenodo.167037). 5) “Feedback section” (4 sec), the gain and the potential loss for that selection were shown on the screen with the sentences “you won XXX” written in green, and “you lost XXX” written in red, and the previous and current amounts of the initial endowment updated. Then, the next trial began again from the top with the “Relax section”. Two practice trials were performed before the proper game, to allow participants to familiarize themselves with the trials’ sequences.

To compute the physiological indexes in line with the SMH, a “pre-choice interval”, corresponding to the 5 seconds before each card selection, was identified. Typically, the pre-choice interval mainly overlapped the “Think” section.

EDA and PPG signals were recorded with a Biopac MP160 (2000 Hz sampling rate) and processed with the Python *“pyphysio”* library [53] to extract physiological indexes. EDA signal was recorded with two reusable electrodes filled with isotonic gel on the first phalanges of the index and middle finger of the non-dominant hand of the participant. EDA signals were then manually inspected to detect noisy signals. Subjects presenting too compromised signals were excluded (*n* = 4).

Due to technical issues on the EDA module, the signals of 45 participants underwent a high-pass filtering stage before the recording. These participants were not included in the analysis. A high-pass (0.05 Hz cutoff-frequency) 3^rd^-order Butterworth filter has been applied to the recorded EDA signals, after downsampling at 128 Hz. aSCR was then computed as the area under the detrended signal during the pre-choice interval and expressed in amplitude units (μsec) per time interval (seconds).

The cardiac activity was recorded through a PPG sensor attached to the first phalange of the thumb finger on the participant’s non-dominant hand. From the PPG signal, InterBeat Intervals (IBI) were extracted. IBI signals for each participant were then visually inspected and corrected or rejected (*n* = 18) when needed. Then, the difference between the average IBI in the pre-choice interval and the average IBI during the “Relax” section was computed (DeltaRRmean) as an index of HR following Priolo et al. [22]. Positive DeltaRRmean values represent greater HR deceleration.

Pupil size changes (Pupil Dilation) were recorded with an eye-tracker Tobii Pro X3-120 system (sampling rate 40 Hz). Pupil Dilation was computed by subtracting the base rate mean pupil size in the Relax section from the mean pupil size measured during the “Think” section for each trial using a Python script. Subjects presenting too comprised data were excluded from the analysis (*n* = 4).

### Analysis plan

Analysis has been conducted following Priolo et al. [22] with the lme4 package [54] on R. A generalized mixed-effect model (GLMM) was used to account for the effect of the experimental conditions on IGT performance (i.e., the probability of selecting from a Good deck) as the task unfolded. Specifically, a logistic two-level multilevel model with subject as random effect was used to test differences in task performance both at individual-level and between conditions. The trial-by-trial unfolding of the task (Trial) was adopted as an independent variable at the first level of the model. To ensure computational stability of the parameter estimation process, the Trial predictor was rescaled from the original range (1,100) to (0.01,1) by dividing the original range by 100 (i.e., maximum number of trials). The dependent variable consisted in a binary vector representing participants’ choice in each trial (Bad deck choice = 0; Good deck choice = 1).

Four models were compared using the likelihood ratio test (LRT), since the models were nested, and the Akaike information criterion (AIC [55]). Specifically, the main effect of the Trial on the task performance was examined in comparison with a first null model accounting only for the mean proportion of selections from the Good decks. Then, the effect of the disgust-eliciting manipulation was assessed by controlling for a possible additional main effect of “Condition” (dummy coded) and the interaction between Trial and Condition. To summarize, the models considered are the following:

- Null Model: Good deck choice ~ 1 + (1 | Subject)
- Trial Main Effect: Good deck choice ~ Trial + (1 + Trial | Subject)
- Trial and Condition Main Effects: Good deck choice ~ Trial + Condition + (1 + Trial | Subject)
- Interaction: Good deck choice ~ Trial + Condition + Trial*Condition + (1 + Trial | Subject)

Results were interpreted considering the coefficient estimates of the winning model in the log-odds scale. Here, p-values are computed by means of a Z-statistic. In addition, a follow-up block-level analysis was performed to further investigate the results of the GLMM, and to provide a deeper understanding of individual behavioral patterns across conditions. Here, the task was divided into 10 equally sized trial blocks to account for the temporal dimension.

Three separate linear mixed effect models, one for each physiological index (aSCR, DeltaRRmean, and Pupil Dilation), with subjects as random effect, were used to assess whether experimental manipulations affected the physiological response differentially for each Condition and type of choice (Good or a Bad deck), across task phases. The same 10 trial blocks used for the behavioral analysis were considered. Participants with an insufficient amount of data points in one block were excluded from the analysis (Final samples: aSCR *n* = 94; DeltaRRMean *n* = 115; Pupil Dilation *n* = 130).

For each physiological index, we performed a model selection within each condition for models including either the main effects of Choice or Trial blocks or the additive/interaction effect of Choice and Trial blocks, as well as a null model where no effects were considered. The model selection was performed by means of the AIC. If the null model was not selected as the best model, potential follow-ups were performed to further investigate the effect(s) related to the winning model.

For the whole corpus of analyses, profile likelihood confidence intervals were considered.

## Results

### Behavioral analysis

Results from model comparison indicated that the model including the interaction between Trial and Condition is the winning model. This model indeed reported a significant reduction of deviance (Chisq(4) = 6.140, *p* = 0.046) and a lower AIC score (Table 1). The presence of a significant interaction between Trail and Condition means that task performance increased as the task unfolded in all conditions but the rate of change of the probability of selecting from a Good deck was affected by the experimental condition.

**Table 1:** Model comparison results.

Model	Parameters	AIC	logLik	Deviance	Chisq.	Df
Null Model	2	17974	-8985	17970		
Trial	5	17521	-8755	17511	62.765 (*p* < .0006)	1
Trial + Condition	7	17523	-8754	17509	1.906 (*p* = .386)	2
Trial × Condition	9	17521	-8751	17503	6.140 (*p* = .046)	2

To better interpret the association between each predictor and task performance, the coefficient estimates of the best model in the log-odd scale were considered (Table 2).

**Table 2:** Coefficient estimates (log-odds scale).

		*Choice*		
Predictors	Log-Odds	CI		*p*
Intercept	-.25	-.44	-.06	.011
Trial	1.06	.65	1.48	< .001
Condition (Congruent)	.06	-.22	.33	.675
Condition (Incongruent)	-.30	-.58	-.02	.038
Trial x Condition (Congruent)	-.25	-.84	.34	.401
Trial x Condition (Incongruent)	.51	-.10	1.12	.099
**Random Effects**				
τ_00_ _Subj_	.28			
τ_11_ _Subj.Trial_percent_ ρ_01_	1.52 -0.57			

In general, the probability of selecting Good decks increased as the task unfolded, as depicted by the significance of the Trial slope (coeff = 1.06, p < .001) thus indicating a learning effect. Further, no significant differences in trend between the *Control* condition (baseline level), and the experimental conditions could be detected. Indeed, only partial evidence for a positive additive effect on the baseline level slope (coeff = .51, p = .099) was shown in the *Incongruent* condition, but it did not however reach significance. Differently, a significant decrease in the intercept in the *Incongruent* condition was found compared to the *Control* condition (coeff = -.30, p < .05). This reflects the fact that individuals in the *Incongruent* condition showed a reduction in selections from the Good decks when they were negatively manipulated in the early stage of the task.

**Fig 1:**
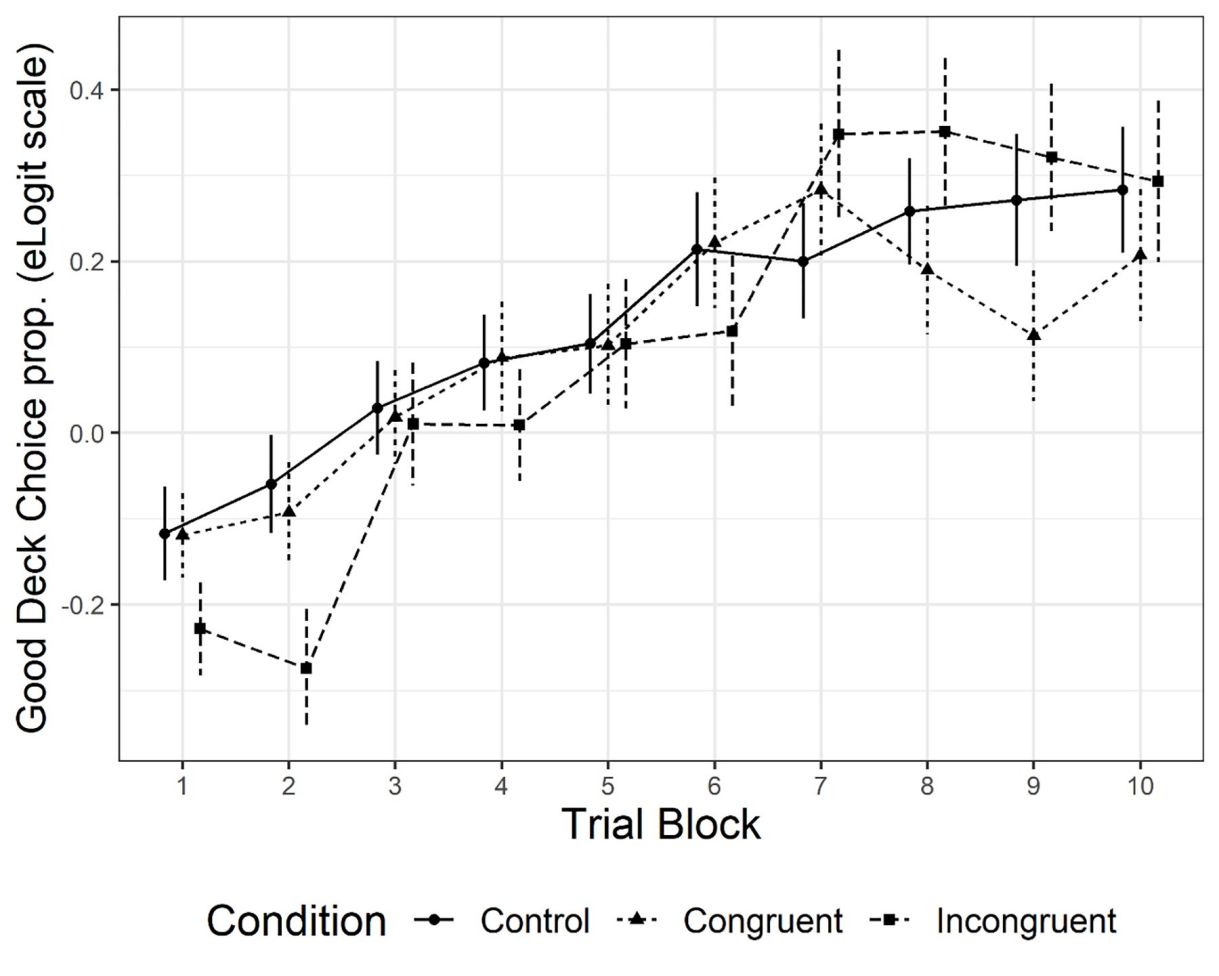
Condition-level Good decks choices (empirical logit scale) as a function of task blocks. Error bars represent standard error.

Taken together, such results are consistent with the view that individual performance was affected by the experimental manipulation in a non-trivial way, by revealing, for instance, different learning behaviors at specific stages of the task. To further investigate this scenario, we performed a task-block analysis, considering 10 trial blocks (10 choices each) for each individual and each Condition as well as the proportion of Good choices for each block as the dependent variable (Fig 1). More precisely, we considered the empirical logit of advantageous choice proportions [56,57]. This transformation allowed to obtain a symmetric distribution of the dependent variable and allowed for a weighted fit of a linear model. Our block analysis wanted to capture higher-order polynomial trends for individual performances and compute block-wise mean differences of Good choices. The main purpose was to detect the task block in which manipulation-related differences in performance occurred.

Four models, namely, a null model, and the interaction between Condition and Task Block for first, second, and third-order polynomial trends were considered. Model comparison results indicated the model with second-order polynomial trend as the winning model. This model indeed reported a significant reduction of deviance (Chisq(6) = 39.05, *p* < .001) and a lower AIC score (Table 3). The fitted (fixed-effect) means are shown in the (S1 Fig in S1 File).

**Table 3:** Model comparison results.

Model	Parameters	AIC	logLik	Deviance	Chisq.	Df
Null Model	3	1175.3	-584.63	1169.26		
First-order	10	1052.0	-516.00	1032.00	137.26(*p* < .001)	7
Second-order	16	1024.9	-496.47	992.94	39.05 (*p* < .001)	6
Third-order	23	1025.6	-489.82	979.64	13.30 (*p* = .0651)	7

**Fig 2:**
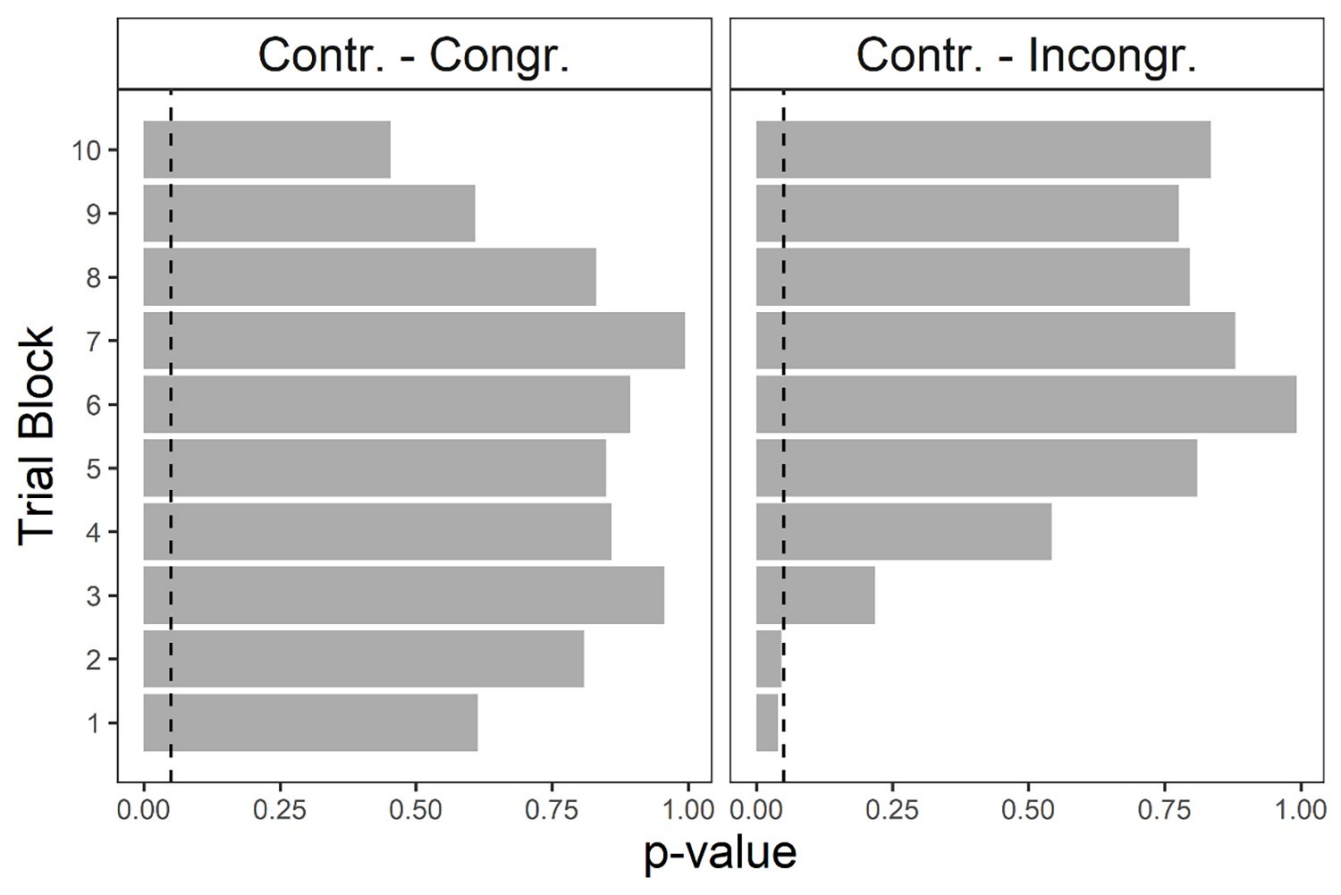
**Control vs. Congruent (left) and Control vs. Incongruent (right) contrast p-values for each task block.** The vertical dotted line indicates the .05 threshold.

The block-wise predicting mean differences were computed for each block separately. In particular, *Control* vs. *Congruent* and *Control* vs. *Incongruent*, block-wise Tukey-corrected contrasts were considered. Results are depicted in Fig 2, where p-values obtained for each contrast are displayed at each block (see the S1 Table in S1 File for the complete table of results).

As can be noted, only differences in performance between *Control* and *Incongruent* conditions can be detected in the first two blocks of the task (block 1, coeff = .104, *p* = .039; block 2, coeff = .076. *p* = .045), thus confirming H1, i.e., that the disgust-eliciting image disrupted participants performance in the *Incongruent* condition in the first stages of the task.

As anticipated in the Introduction, other potential strategies from the one proposed by Bechara et al., were found to be used by non-impaired participants, e.g., some studies indicated that participants might prefer decks with low frequencies of losses [29,30]. We thus controlled for a potential effect of our disgusting manipulation on the selection of advantageous choice alternatives (i.e., Deck C and Decks D), by distinguishing between high (decks D) and low (deck C) frequency loss decks. However, no significant results emerged from this analysis (full results can be found in the Supporting Information, Section 2).

### Physiological analysis

**Fig 3:**
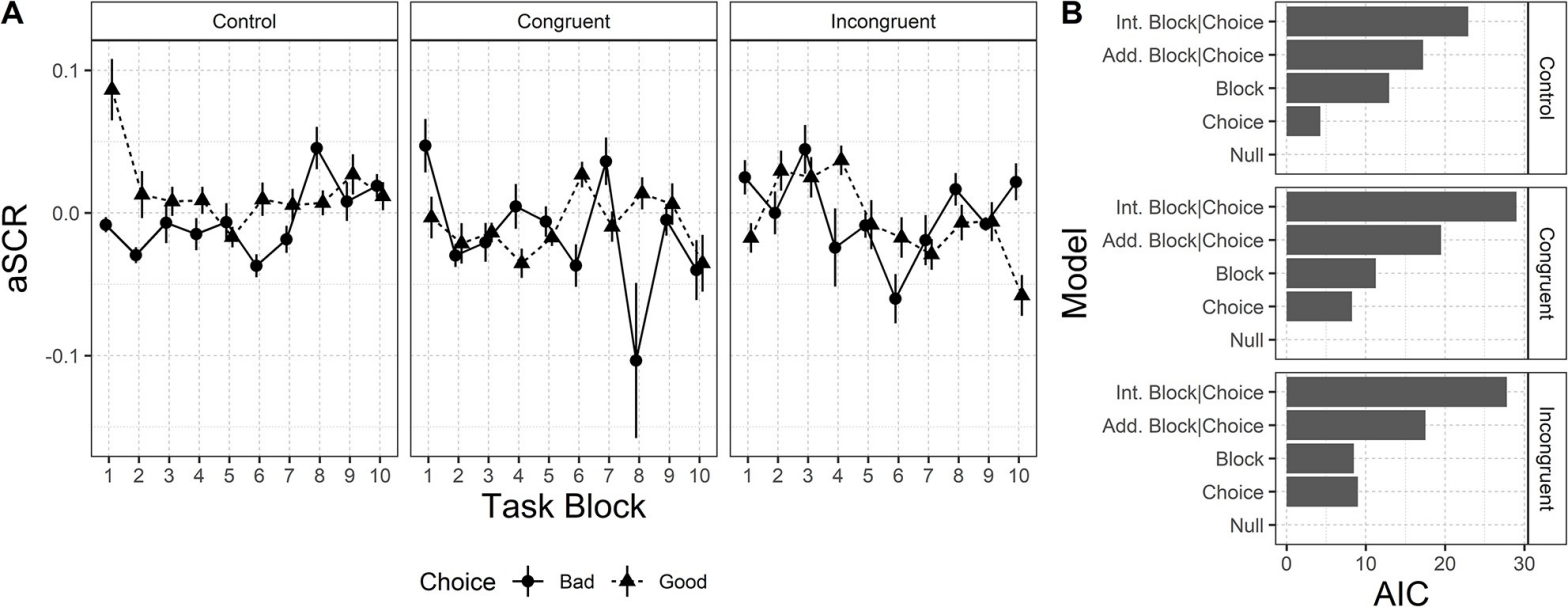
The average value of the aSCR for each condition and the AIC values for the five models considered. Panel A. aSCR pattern for each condition, in relation to each choice, across task blocks. Panel B. AIC values for the five models considered (AIC is rescaled for visualization purposes by subtracting the AIC of the preferred model, i.e., the lowest value, to the others). Error bars represent standard error.

*aSCR*. Results for aSCR are depicted in Fig 3. Panel A shows the average value of the aSCR during the unfolding of the task blocks. Panel B shows the results of the model selection procedure. The null model is preferred across all the conditions, suggesting no increasing/decreasing across-block patterns or between-choice differences. Results suggest that within each condition, aSCR is not modulated by the choice, or by the unfolding of the task.

**Fig 4:**
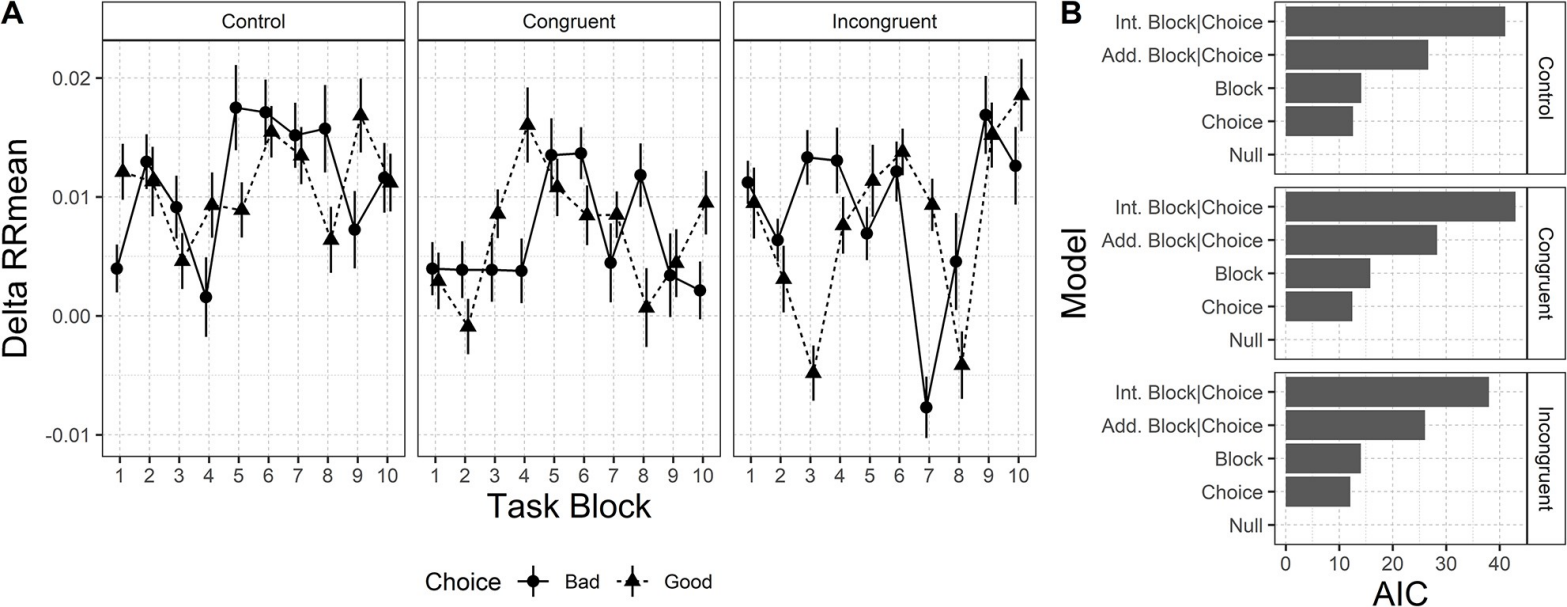
DeltaRRMean pattern for each condition, and AIC values for the five models considered. Panel A. DeltaRRMean pattern for each condition, in relation to each choice, across task blocks. Panel B. AIC values for the five models considered (AIC is rescaled for visualization purposes by subtracting the AIC of the preferred model, i.e. the lowest value, to the others). Error bars represent standard error.

*Cardiac activity*. Results for cardiac activity are depicted in Fig 4. Panel A shows the average value of the DeltaRRMean during the unfolding of the task blocks. Panel B shows the results of the model selection procedure. Even in this case, results suggest that within each condition, DeltaRRMean is not modulated by the choice, or by the unfolding of the task. The null model is preferred for each condition.

**Fig 5:**
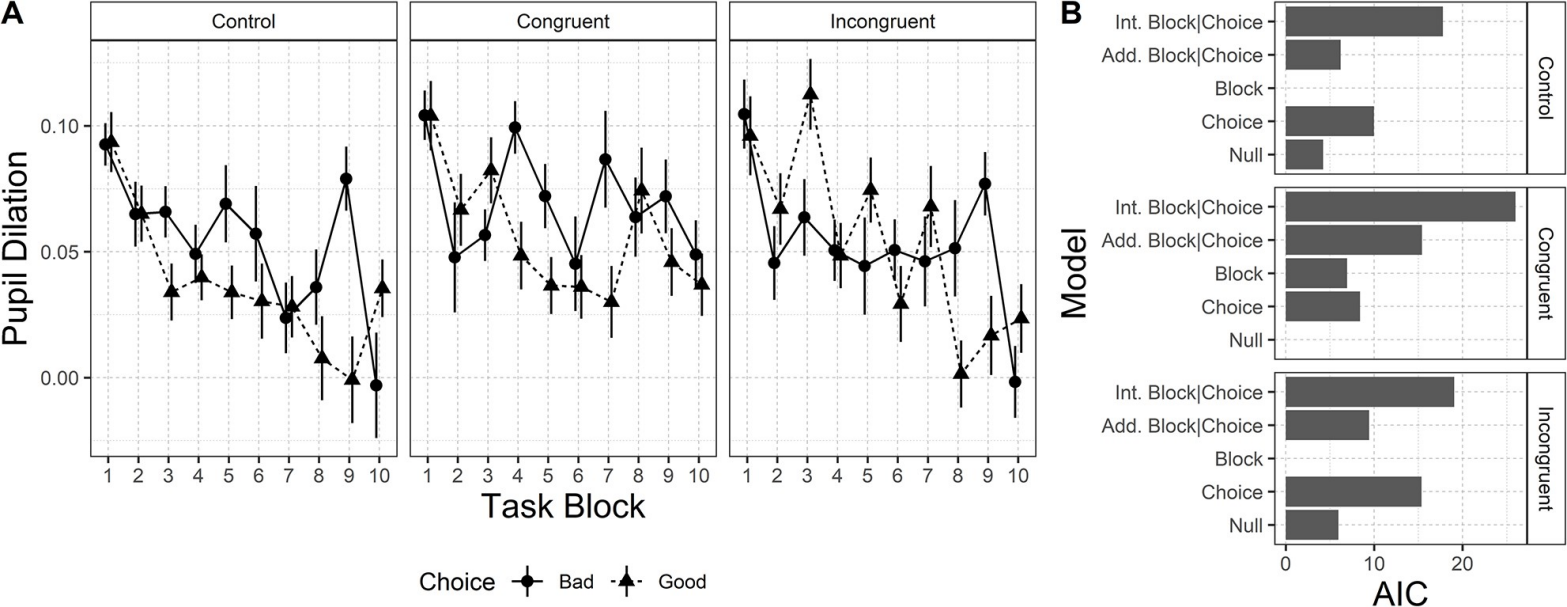
Pupil Dilation pattern for each condition, and AIC values for the five models considered. Panel A. Pupil Dilation pattern for each condition, in relation to each choice, across task blocks. Panel B. AIC values for the five models considered (AIC is rescaled for visualization purposes by subtracting the AIC of the preferred model, i.e. the lowest value, to the others). Error bars represent standard error.

*Pupil Dilation*. Results for pupil dilation are depicted in Fig 5. Panel A shows the average value of the Pupil Dilation during the unfolding of the task blocks. Panel B shows the results of the model selection procedure. The null model is preferred only in the Congruent condition, suggesting that neither the task blocks nor the choice modulate the physiological signal in this condition. On the contrary, the model including only the main effect of the task block is selected as the best model in both Control and Incongruent conditions. A follow-up mixed-effect model analysis showed a significant negative trend in both conditions (Control, coef. = -.006, t(828) = -4.17, *p* <0.001; Incongruent, coef. = -.007, t(722) = -4.27, *p* <0.001). Together these results suggest that Pupil Dilation decreases progressively as the task unfolds, without showing any difference between Good or Bad deck choices.

Finally, previous studies showed that individual differences in personality traits (i.e., impulsivity) and state mood can mediate IGT performances, although results are not always consistent [22,58–62]. We thus controlled for the effect of individual differences, as well as the one of demographic information (i.e., age and gender) in our study too. Nevertheless, no significant effect of any of the covariates could be detected. Full methods, results, and discussion on the (missing) effect of individual differences in our study can be found in the Supporting Information, Section 3.

## Discussion

In this study, we investigated whether an incidental, normatively irrelevant emotional reaction of disgust could influence participants’ performance in a manipulated IGT. Following Priolo et al. [22] the presentation of a disgust-eliciting image was associated with selections from Good (*Incongruent* condition) or Bad decks (*Congruent* condition). We expected that participants in the experimental conditions would have experienced two reactions: a first normatively relevant affective reaction driven by the disadvantageous risk contingency of the Bad decks, and a second normatively irrelevant emotional one driven by the disgust-eliciting image. Consequently, drawing on the “risk as feeling” framework [7] and the SMH [8,9], we expected that the presence of the disgust-eliciting image would have led participants to avoid the manipulated decks, thus having a detrimental effect in the *Incongruent* condition and a beneficial one in the *Congruent* condition.

In line with previous studies [8,13–15,22–24,40], a learning effect (i.e., increased probability of selecting from a Good deck as the task unfolded) was found in all conditions. However, this learning effect was modulated by the experimental manipulation as shown by a significant interaction between the Trial (i.e., unfolding of the task) and Condition variables.

Specifically, a significant decrease in the intercept between *Incongruent* and *Control* conditions was found, confirming our hypothesis of a detrimental effect of the disgust-eliciting image in this condition (H1) in line with previous studies [22–24,40]. Moreover, this detrimental effect was found to be limited to the early stages of the task (i.e., first twenty trials) as revealed by the block analysis in line with Pittig et al. [23]. No significant differences in the probability of selecting from a Good deck were found from the third task block. We could thus speculate that at the beginning of the task, when the risk contingency of the decks is still obscure, the irrelevant emotional reaction of disgust has been effective in disrupting participants’ performance. As the task unfolded and the decks’ risk contingency got clearer instead, the mismatch between the relevant affective reaction driven by Good decks’ advantageousness and the irrelevant emotional one elicited by the disgusting image in the *Incongruent* condition got clearer too. Participants might thus have gradually learned to endure the disgusting reaction and correct their performance.

No differences in performance trends have been instead detected between *Congruent* and *Control* conditions, thus rejecting H2. This result is in contrast with Pittig et al. [23] in which a beneficial effect (i.e., lower selections from the Bad decks and higher selections from the Good ones) was detected in their Congruent condition. Nevertheless, this result is in line with the asymmetrical effect detected among conditions in Priolo et al. [22] and can be interpreted as a discounting effect, i.e., people’s tendency to discount ambiguous information. In a series of studies proposed by van Dijk & Zeelenberg [63] for instance, participants presented with ambiguous information tended to discount it and reported similar performances as those that were presented with no information at all (Control). We can speculate that participants in this study and Priolo et al. [22] might have similarly perceived the experimental manipulation as ambiguous and not informative (i.e., as actually normatively irrelevant) and decided to discount it, thus performing as those in the *Control* condition. The fear-inducing manipulation used in Pittig et al. [23] instead, could not be discounted since it was related to participants’ phobia, and thus meaningful and not ambiguous to them.

Moreover, our trend analysis showed also that towards the end of the game (Block 8–10) the variance in the proportion of choice selection was still generally high between conditions. This finding is interesting and intriguing and can only be partially explained by the potential interference of our disgusting manipulation on the learning process. Nevertheless, this finding is in line with Steingroever et al. [18] claim of the absence of an “exploration-exploitation trade-off” as originally suggested by Bechara et al., as their metanalytic finding showed that many participants keep switching between decks thus suggesting that 100 trials might not be sufficient to learn about the nature of the task.

Drawing on the SMH, we also expected to detect the effect of the disgust-eliciting manipulation from a physiological point of view (i.e., anticipatory autonomic activation). Specifically, we expected that the presence of the two competing affective reactions in the *Incongruent* condition would have misled participants also at a somatic level preventing their somatic marker system from developing a clear somatic alert towards the Bad decks.

Results showed that aSCR and cardiac activity trends before selections from any decks did not vary in the *Incongruent* condition. This finding confirms the hypothesis that the disgust-eliciting manipulation would have impeded participants in this condition from associating clear and useful somatic markers to the decks (H3).

Similarly, no differences in anticipatory physiological response between the decks could be detected in the *Congruent* condition. This finding is in contrast with our expectation of detecting a higher autonomic activation before selections from the Bad decks in this condition (H4).

The absence of physiological activation as the task unfolds, especially in terms of enhanced aSCR prior to selection from Bad decks, is in contrast with previous studies [22,45,64] and Bechara and Damasio’s assumption of the generation of somatic markers [9,16]. Nevertheless, studies assessing the activation of somatic markers at the cardiac level are still very sparse and with mixed results, while recent metanalyses highlighted that the effects found for aSCR activation are only small and potentially misled by publication bias and heterogeneity of results [43,44]. Overall, our results highlight once again the necessity of further investigations on the generation of somatic markers and their influence on IGT performance in order to truly understand this phenomenon correctly.

Moreover, a decreasing trend of pupil dilation as associated with task unfolding was detected in both *Control* and *Incongruent* conditions. These results are interesting since, to the best of our knowledge, no previous studies focused on pupil dilation’s trend as a function of IGT unfolding and as a somatic marker. We can thus only speculate about the meaning of our results. It is possible that this progressive reduction in pupil dilation might be an effect of participants’ habituation to the task. Previous studies indeed associated decreasing pupil dilation to task habituation although with mixed findings [65–67]. Habituation to disgusting stimuli is also well known in the literature, even though only a few studies investigated this effect with physiological measures, and those who did, did not include a measurement of pupil dilation [68,69]. Moreover, most of the studies on the habituation effect focused on pupil reaction to feedback or emotional stimuli, while studies investigating pupil reaction *before* the stimulus presentation as in our design are lacking as well as studies using the IGT specifically, thus making comparisons difficult. Moreover, the absence of an effect in the *Congruent* condition remains troublesome and calls for further investigations to disentangle the role of pupil dilation in IGT performance.

Overall, the fact that participants in the experimental conditions performed similarly to those in the *Control* condition and the absence of somatic markers generation in our study is in line with Maia & McClelland’s and other critiques of the IGT [18,19,70,71]. According to the authors indeed, decks’ gain/loss schedule might be more cognitively penetrable than supposed by Bechara and colleagues. Participants might indeed develop explicit knowledge of the decks’ contingencies earlier than expected thus making the generation of somatic markers useless to reach a good performance, which is indeed reached through a more cognitive rather than emotional strategy. This thesis seems to be in line especially with our findings from the *Congruent* condition in which participants performed similarly to the ones in the *Control* condition even without developing any somatic markers and potentially discounting the emotional reaction of disgust that could have instead helped them to pursue an even more advantageous strategy.

### Limitations and future directions

As stated above, it is possible that participants in this study discounted the emotional reaction driven by the disgust-eliciting image and used a more cognitive strategy. Nevertheless, we did not ask participants about the actual strategy they used to perform the task nor how they interpreted and used the disgust-eliciting manipulation. Future studies should try to validate our speculations by explicitly assessing participants’ strategy and manipulation interpretation at the end of the game or at every set of twenty picks [13].

Moreover, a single disgust-eliciting image was used in this study. This choice was made in line with previous studies [23,24,40] and to avoid confounding effects potentially arising from using multiple images. This, however, might limit the generalization of our findings to other stimuli. Furthermore, although the image used was rated as extremely disgusting and was thus highly unpleasant to see, we cannot rule out a habituation effect that might have been associated with the decreasing trend in pupil dilation detected in *Incongruent* condition. This effect might partially explain why the effect of the manipulation was limited to the initial trials. Future studies should thus consider using multiple images with similar disgust ratings or mixing images with different ones to check for potential habituation effects and increase the generalizability of the present findings.

Finally, also the role of positive emotional reactions might be worth investigating. It is possible that joyful or pleasant reactions might be able to mask Bad decks’ disadvantageousness in a similar way as the negative ones do with Good ones.

## Conclusions

In this study we showed how a normatively irrelevant emotional reaction of disgust can impair decision-making. However, this effect is limited to situations in which the emotional reaction does not match with normative characteristics of the options and uncertainty is high. When uncertainty is reduced and when the normatively irrelevant emotional reaction and the normative characteristics of the option do match instead, people seem to discount the negative emotional information, and the effect of the experimental manipulation is reduced.

## Supporting information

S1 File (DOCX)
