# Supplementary material for: Normatively irrelevant disgust interferes with decision under uncertainty: Insights from the Iowa gambling task
Source: PLoS One. 2024 Aug 1;19(8):e0306689. doi: 10.1371/journal.pone.0306689 (PMC11293706; doi:10.1371/journal.pone.0306689)
Supplement: S1 File — (DOCX) [file pone.0306689.s001.docx]

**Supporting Information for the article “Normatively irrelevant disgust interferes with decision under uncertainty: insights from the Iowa Gambling Task”**

**Section 1 Additional analysis**

In what follows, we provide Tables for analytic outputs presented in the main paper.

**S1 Fig. Condition-level fitted mean of Good decks choices (empirical logit scale) as a function of task blocks**

*
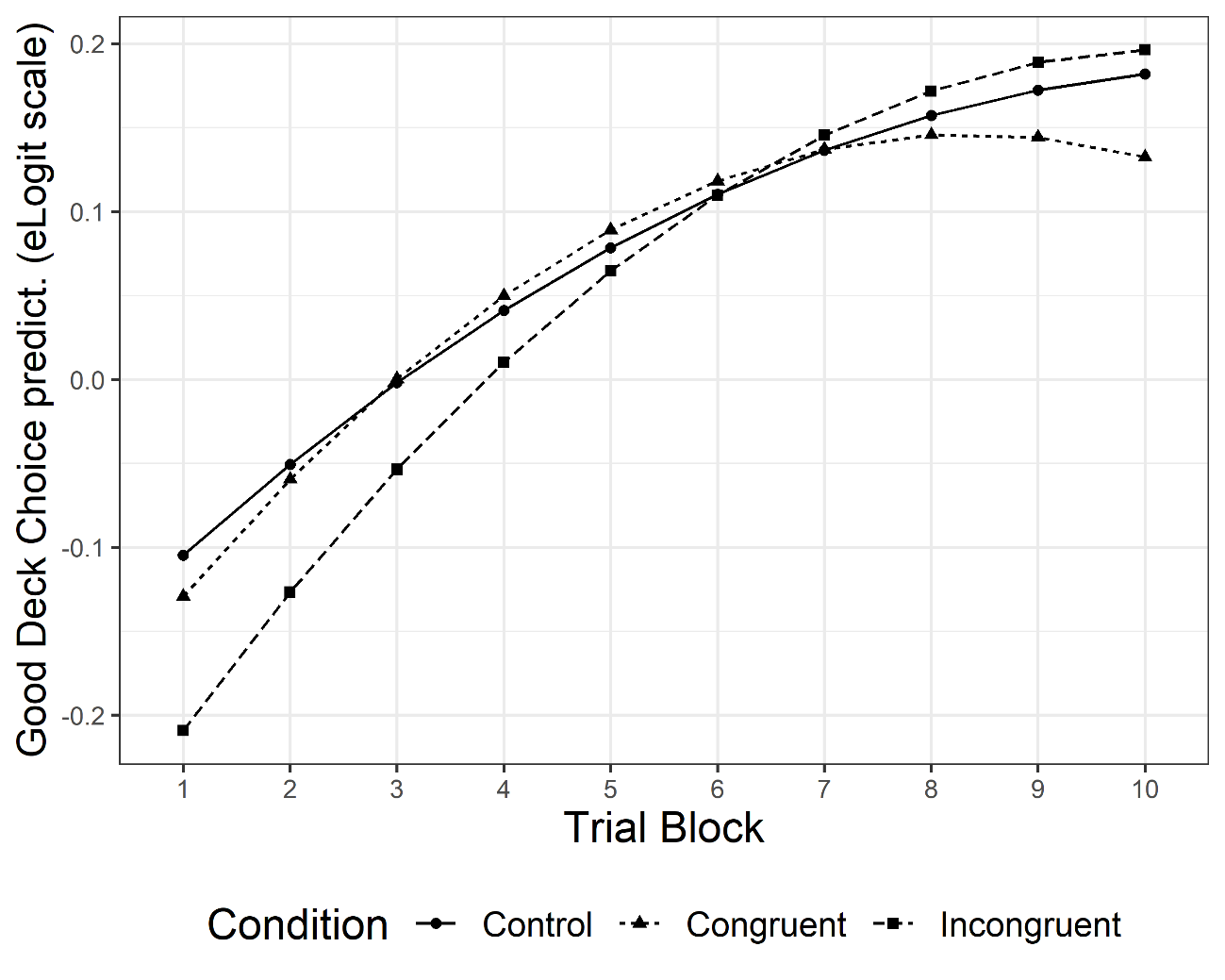
*

Below, the block-wise contrast analysis results (Table S1), marginal condition-level fixed-effect slopes for Good and Bad deck choices related to anticipatory SCR (Table S2), DeltaRRmean (Table S3), Pupil Dilation (Table S4), and trend contrast analysis for Pupil Dilation (Table S5, S6 and S 7) are shown.

**S1 Table. Results of the block-wise contrast analysis.**

| **Contrast** | **Block** | **Estimate** | **SE** | ***t-ratio*** | ***p-value*** |
| --- | --- | --- | --- | --- | --- |
| Control-Congruent | 1 | 0.0246253 | 0.0487505 | .505 | .614 |
| Control–Incongruent | 1 | 0.1045404 | 0.0506553 | 2.064 | .039 |
| Control-Congruent | 2 | 0.0088812 | 0.0367707 | .242 | .809 |
| Control–Incongruent | 2 | 0.0761712 | 0.0380474 | 2.002 | .046 |
| Control-Congruent | 3 | -0.022352 | 0.0406277 | -.055 | .956 |
| Control–Incongruent | 3 | 0.0515897 | 0.0417989 | 1.234 | .218 |
| Control-Congruent | 4 | -0.0087239 | 0.0492176 | -.177 | .860 |
| Control–Incongruent | 4 | 0.0307958 | 0.0505553 | .609 | .543 |
| Control-Congruent | 5 | -0.0105848 | 0.0556324 | -.190 | .850 |
| Control–Incongruent | 5 | 0.0137895 | 0.0571242 | .241 | .809 |
| Control-Congruent | 6 | -0.0078180 | 0.0580808 | -.135 | .893 |
| Control–Incongruent | 6 | 0.0005709 | 0.0596401 | .010 | .992 |
| Control-Congruent | 7 | -0.0004235 | 0.0569209 | -.007 | .994 |
| Control–Incongruent | 7 | -0.0088601 | 0.0585079 | -.151 | .880 |
| Control-Congruent | 8 | 0.0115988 | 0.0543562 | .231 | .831 |
| Control–Incongruent | 8 | -0.0145034 | 0.0560909 | -.259 | .796 |
| Control-Congruent | 9 | 0.0282488 | 0.0552664 | .511 | .610 |
| Control–Incongruent | 9 | -0.0163592 | 0.0575193 | -.284 | .776 |
| Control-Congruent | 10 | 0.0495266 | 0.0659842 | .751 | .453 |
| Control–Incongruent | 10 | -0.0144273 | 0.0962165 | -.208 | .835 |

**S2 Table. Marginal condition-level fixed-effect slopes for Good and Bad deck choices related to anticipatory SCR.**

| **Deck choice** | **Condition** | **Slope** | **SE** | **0.025 CI** | **0.0975 CI** |
| --- | --- | --- | --- | --- | --- |
| Bad | Control | .0082764 | .0082647 | -.0079542 | .0245070 |
| Good | Control | -.0237732 | .0081937 | -.0398644 | -.0076819 |
| Bad | Congruent | -.0165964 | .0084106 | -.0331135 | -.0000793 |
| Good | Congruent | -.0003781 | .0083906 | -.0168665 | .0161102 |
| Bad | Incongruent | -.0103667 | .0085494 | -.0271564 | .0064231 |
| Good | Incongruent | -.0021040 | .0085502 | -.0188952 | .0146871 |

**S3 Table. Marginal condition-level fixed-effect slopes for Good and Bad deck choices related to DeltaRRmean.**

| **Deck choice** | **Condition** | **Slope** | **SE** | **0.025 CI** | **0.0975 CI** |
| --- | --- | --- | --- | --- | --- |
| Bad | Control | .0021890 | .0014359 | -.0006298 | .0050077 |
| Good | Control | -.003280 | .0014350 | -.0031450 | .0024889 |
| Bad | Congruent | .0009984 | .0013908 | -.0017318 | .0037286 |
| Good | Congruent | .0024513 | .0013889 | -.0002750 | .0051777 |
| Bad | Incongruent | -.0009549 | .0014559 | -.0038128 | .0019030 |
| Good | Incongruent | .0002217 | .0014580 | -.0026403 | .0030838 |

**S4 Table. Marginal condition-level fixed-effect slopes for Good and Bad deck choices related to Pupil Dilation.**

| **Deck choice** | **Condition** | **Slope** | **SE** | **0.025 CI** | **0.0975 CI** |
| --- | --- | --- | --- | --- | --- |
| Bad | Control | -.0179598 | .0073120 | -.0323106 | -.0036089 |
| Good | Control | -.0257083 | .0073034 | -.0400424 | -.0113743 |
| Bad | Congruent | -.0112434 | .0075249 | -.0260121 | .0035254 |
| Good | Congruent | -.0224972 | .0075239 | -.0372640 | -.0077304 |
| Bad | Incongruent | -.0207798 | .0077519 | -.0359940 | -.0055655 |
| Good | Incongruent | -.0205183 | .0077689 | -.0357660 | -.0052707 |

**S5 Table. Results of paired-contrast analysis of Pupil Dilation trends between type of Deck in the three conditions.**

| **Contrast** | **Condition** | **Estimate** | **SE** | ***t*. ratio** | ***p*. value** |
| --- | --- | --- | --- | --- | --- |
| Bad–Good | Control | .007749 | .0103 | 1.100 | .45 |
| Bad–Good | Congruent | .011254 | .0106 | .644 | .29 |
| Bad–Good | Incongruent | -.000261 | .0110 | 1.345 | .98 |

**S6 Table. Condition-level contrast analysis for trends related to Good and Bad deck choices for Pupil Dilation.**

| **Deck choice** | **Condition** | **Slope Diff.** | **SE** | **0.025 CI** | **0.0975 CI** |
| --- | --- | --- | --- | --- | --- |
| Bad - Good | Control | .0077486 | .0103348 | .07497531 | .4536031 |
| Bad - Good | Congruent | .0112539 | .0106415 | 1.0575486 | .2905505 |
| Bad - Good | Incongruent | -.0002614 | .0109737 | -.0238221 | .9809999 |

**Section 2 Deck Analysis**

In this section we separately examined the effect of experimental manipulation on the selection of advantageous choice alternatives, by distinguishing between high and low frequency loss decks. In particular, we performed the same task block analysis performed in the main manuscript, for every single advantageous deck (C and D in Figure S2), to detect preferential effect of Condition on the deck presenting either low or high loss frequency.


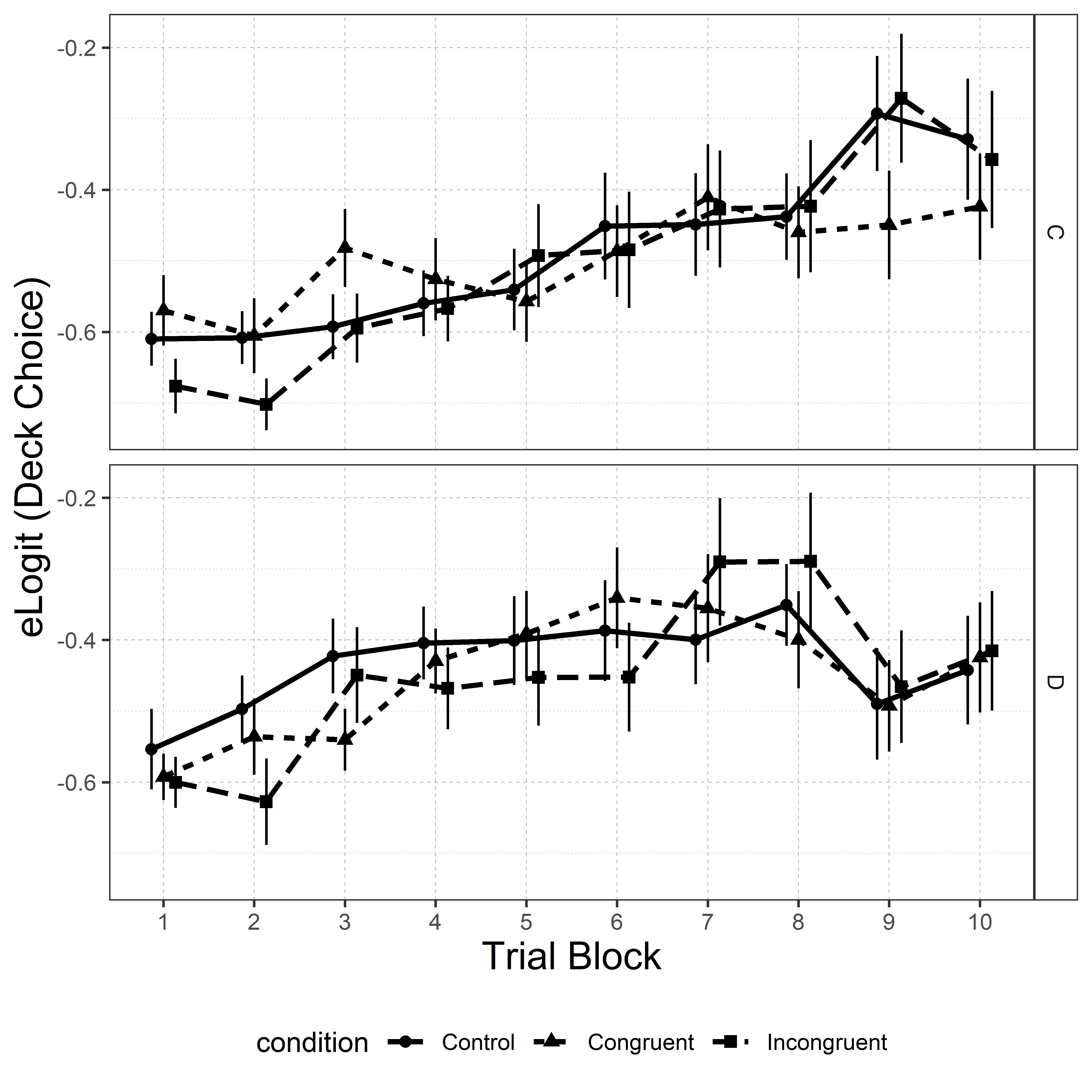


**S2 Fig. Condition-level advantageous deck choices (empirical logit scale) as a function of task blocks. C and D panels represent high-frequency and low-frequency loss decks, respectively.**

## Even in this case, a first model selection was performed to verify the presence of higher-order polynomial trend in deck choices. Four models, namely, a null model, and the interaction between Condition and Task Block for first, second, and third-order polynomial trends were considered. Model comparison results indicated the model with third-order polynomial trend as the winning model for both decks. However, a follow-up analysis could not detected block-level differences in deck choices between the different conditions (Table S7).

**S7 Table. Deck C. Condition-level contrast analysis for trends related to Good and Bad deck choices.**

| contrast | p.value | Block |
| --- | --- | --- |
| Contr. - Congr. | 0.8690218 | 1 |
| Contr. - Incongr. | 0.2386491 | 1 |
| Contr. - Congr. | 0.8523507 | 2 |
| Contr. - Incongr. | 0.2171863 | 2 |
| Contr. - Congr. | 0.8252585 | 3 |
| Contr. - Incongr. | 0.4557595 | 3 |
| Contr. - Congr. | 0.9535641 | 4 |
| Contr. - Incongr. | 0.6879183 | 4 |
| Contr. - Congr. | 0.8279252 | 5 |
| Contr. - Incongr. | 0.9138088 | 5 |
| Contr. - Congr. | 0.6300402 | 6 |
| Contr. - Incongr. | 0.9120362 | 6 |
| Contr. - Congr. | 0.4955934 | 7 |
| Contr. - Incongr. | 0.8101747 | 7 |
| Contr. - Congr. | 0.3900646 | 8 |
| Contr. - Incongr. | 0.7453861 | 8 |
| Contr. - Congr. | 0.3329570 | 9 |
| Contr. - Incongr. | 0.7170881 | 9 |
| Contr. - Congr. | 0.4908547 | 10 |

**S8 Table. Deck D. Condition-level contrast analysis for trends related to Good and Bad deck choices.**

| contrast | p.value | Block |
| --- | --- | --- |
| Contr. - Congr. | 0.4923855 | 1 |
| Contr. - Incongr. | 0.4910527 | 1 |
| Contr. - Congr. | 0.3610677 | 2 |
| Contr. - Incongr. | 0.1961875 | 2 |
| Contr. - Congr. | 0.5981447 | 3 |
| Contr. - Incongr. | 0.3401651 | 3 |
| Contr. - Congr. | 0.8508585 | 4 |
| Contr. - Incongr. | 0.6306296 | 4 |
| Contr. - Congr. | 0.9232210 | 5 |
| Contr. - Incongr. | 0.9780948 | 5 |
| Contr. - Congr. | 0.7695033 | 6 |
| Contr. - Incongr. | 0.6559949 | 6 |
| Contr. - Congr. | 0.6706816 | 7 |
| Contr. - Incongr. | 0.4528362 | 7 |
| Contr. - Congr. | 0.5700544 | 8 |
| Contr. - Incongr. | 0.2890158 | 8 |
| Contr. - Congr. | 0.4981724 | 9 |
| Contr. - Incongr. | 0.2009569 | 9 |
| Contr. - Congr. | 0.7179300 | 10 |
| Contr. - Incongr. | 0.5159635 | 10 |

**Section 3 Individual differences**

Previous studies showed that individual differences in impulsivity traits and state mood and demographics can influence IGT performances, although with mixed results (Buelow & Suhr, 2009). For instance associations between riskier performances in the IGT and impulsivity and sensations-seeking personality traits have been found (Buelow & Suhr, 2009; Priolo et al., 2021; Suhr & Tsanadis, 2007). Specifically, disruptive performances have been associated with the BAS components of the BIS/BAS scales (Carver & White, 1994). Moreover, negative state mood is known to increase likelihood estimates for negative events and risk-taking (Bruyneel et al., 2009; Johnson & Tversky, 1983; Mohanty & Suar, 2014). Previous studies indeed associated negative affect at the moment of the task administration with riskier performances at the IGT in both clinical and non-clinical populations (Buelow & Suhr, 2009, 2013; Must et al., 2006; Priolo et al., 2021; Suhr & Tsanadis, 2007).

Thus, in the present study effects of individual differences in impulsivity traits and negative state mood have been also controlled for along with the one of demographic information (i.e., age and gender). The PANAS scale (Watson et al., 1988) was used to assess subjects’ pre- and post-task state mood. Participants rated on a 5-point scale to what extent their current mood was represented by a list of 20 positive and negative emotional states. Only the negative mood subscale was used in the analysis.

To assess participants' impulsivity traits, the Italian validated version of the BIS/BAS scale (Carver & White, 1994; Leone et al., 2002) was used. Participants were asked to rate on a 5-point scale to what extent 7 BIS scale items and 13 BAS scales items better described themselves. The BAS scale can be divided into three subscales (Drive, Reward Responsiveness, and Fun Seeking) representing different facets of the BAS system. Since previous studies associated the Fun Seeking scale with IGT performance and impulsive personality (Buelow & Suhr, 2009; Priolo et al., 2021; Suhr & Tsanadis, 2007), the analyses focused on that subscale only.

The analysis consisted of the generalized linear mixed-effects winning model in the main paper, that is, the model with the interaction between Condition and Trial. Additionally, the covariates were included together with Age and Gender information. Regression coefficients related to covariates affect individual-level intercepts. Results from the analysis are shown in Table S3. As can be noted, covariates are not significant.

This result contrasts our expectations and previous literature (e.g., Buelow & Suhr, 2009; Peters & Slovic, 2000; Suhr & Tsanadis, 2007), but is in line with other previous studies that also failed to find significant associations between (negative) state mood, personality traits or demographic variables and IGT performance (e.g., Brand et al., 2007; Brand & Altstötter-Gleich, 2008; Suhr & Hammers, 2010). Overall, these findings highlight once again the presence of mixed results and the lack of a clear understanding of the influence of individual differences on IGT performance. Further studies aimed at addressing this issue are thus necessary.

**S9 Table. Results of the GLMM with covariates.**

|  |  | *Choice* | |  |
| --- | --- | --- | --- | --- |
| **Predictors** | **Log-Odds** | **CI** | | ***p*** |
| Intercept | -.24 | -.45 | -.03 | **.027** |
| Trial | 1.06 | .65 | -1.48 | **<.001** |
| Condition (Congruent) | .07 | -.21 | -.34 | .647 |
| Condition (Incongruent)  Panas Negative  BIS  Fun Seeking  Age  Gender (Male) | -.32  .00  .01  -.00  .00  -.01 | -.60  -.02  -.01  -.03  -.01  -.20 | -.03  .03  .04  .03  .02  .17 | **.029**  .762  .324  .999  .630  .891 |
| Trial x Condition (Congruent) | -.25 | -.84 | .34 | .402 |
| Trial x Condition (Incongruent) | .51 | -.10 | 1.12 | .099 |
|  |  |  |  |  |
| **Random Effects** |  |  |  |  |
| σ^2^ | 3.29 |  |  |  |
| τ_00_ _Subj_ | .28 |  |  |  |
| τ_11_ _Subj.Trial_percent_ | 1.52 |  |  |  |

**References**

Brand, M., & Altstötter-Gleich, C. (2008). Personality and decision-making in laboratory gambling tasks – Evidence for a relationship between deciding advantageously under risk conditions and perfectionism. *Personality and Individual Differences*, *45*(3), 226–231. https://doi.org/10.1016/j.paid.2008.04.003

Brand, M., Recknor, E. C., Grabenhorst, F., & Bechara, A. (2007). Decisions under ambiguity and decisions under risk: Correlations with executive functions and comparisons of two different gambling tasks with implicit and explicit rules. *Journal of Clinical and Experimental Neuropsychology*, *29*(1), 86–99. https://doi.org/10.1080/13803390500507196

Bruyneel, S. D., Dewitte, S., Franses, P. H., & Dekimpe, M. G. (2009). I felt low and my purse feels light: Depleting mood regulation attempts affect risk decision making. *Journal of Behavioral Decision Making*, *22*(2), 153–170. https://doi.org/10.1002/bdm.619

Buelow, M. T., & Suhr, J. A. (2009). Construct Validity of the Iowa Gambling Task. *Neuropsychology Review*, *19*(1), 102–114. https://doi.org/10.1007/s11065-009-9083-4

Buelow, M. T., & Suhr, J. A. (2013). Personality characteristics and state mood influence individual deck selections on the Iowa Gambling Task. *Personality and Individual Differences*, *54*(5), 593–597. https://doi.org/10.1016/j.paid.2012.11.019

Carver, C. S., & White, T. L. (1994). Behavioral inhibition, behavioral activation, and affective responses to impending reward and punishment: The BIS/BAS Scales. *Journal of Personality and Social Psychology*, *67*(2), 319–333. https://doi.org/10.1037/0022-3514.67.2.319

Johnson, E. J., & Tversky, A. (1983). Affect, generalization, and the perception of risk. *Journal of Personality and Social Psychology*, *45*(1), 20–31. https://doi.org/10.1037/0022-3514.45.1.20

Leone, L., Pierro, A., Mannetti, L., & Leone, L. (2002). Validità della versione italiana delle scale BIS/BAS di Carver e White (1994): Generalizzabilità della struttura e relazioni con costrutti affini. *Giornale Italiano Di Psicologia*, *2/2002*. https://doi.org/10.1421/1245

Mohanty, S. N., & Suar, D. (2014). Decision Making under Uncertainty and Information Processing in Positive and Negative Mood States. *Psychological Reports*, *115*(1), 91–105. https://doi.org/10.2466/20.04.PR0.115c16z2

Must, A., Szabó, Z., Bódi, N., Szász, A., Janka, Z., & Kéri, S. (2006). Sensitivity to reward and punishment and the prefrontal cortex in major depression. *Journal of Affective Disorders*, *90*(2), 209–215. https://doi.org/10.1016/j.jad.2005.12.005

Peters, E., & Slovic, P. (2000). The Springs of Action: Affective and Analytical Information Processing in Choice. *Personality and Social Psychology Bulletin*, *26*(12), 1465–1475. https://doi.org/10.1177/01461672002612002

Priolo, G., D’Alessandro, M., Bizzego, A., & Bonini, N. (2021). Normatively Irrelevant Affective Cues Affect Risk-Taking under Uncertainty: Insights from the Iowa Gambling Task (IGT), Skin Conductance Response, and Heart Rate Variability. *Brain Sciences*, *11*(3), Article 3. https://doi.org/10.3390/brainsci11030336

Suhr, J. A., & Tsanadis, J. (2007). Affect and personality correlates of the Iowa Gambling Task. *Personality and Individual Differences*, *43*(1), 27–36. https://doi.org/10.1016/j.paid.2006.11.004

Suhr, J., & Hammers, D. (2010). Who Fails the Iowa Gambling Test (IGT)? Personality, Neuropsychological, and Near-Infrared Spectroscopy Findings in Healthy Young Controls. *Archives of Clinical Neuropsychology*, *25*(4), 293–302. https://doi.org/10.1093/arclin/acq017

Watson, D., Clark, L. A., & Tellegen, A. (1988). Development and validation of brief measures of positive and negative affect: The PANAS scales. *Journal of Personality and Social Psychology*, *54*(6), 1063–1070. https://doi.org/10.1037//0022-3514.54.6.1063
